# Molecular genetics and long-term outcomes of primary distal renal tubular acidosis in Asia

**DOI:** 10.1093/ndt/gfaf222

**Published:** 2025-10-24

**Authors:** Sayali Thakare, Anurag Lila, Vaibhav Keskar, Thenral Geetha, Kruteesh Kumar BS, Nikhil Rao, Ashwini Joga, Prateek Shirke, Abhimanyu Sardar, Rita Bothara, Prajakta Kadu, Geetanjali Thakur, Tulsi Modi, Sreyashi Bose, Divya Bajpai, Tejashree More, Prabhakar Kedar, Bhagyshree Deshmukh, Nanda Pai, Pratap Jadhav, Tushar Bandgar, Niwrutti Hase, Tukaram Jamale

**Affiliations:** Department of Nephrology, Seth GSMC and KEM Hospital, Mumbai, India; Department of Endocrinology, Seth GSMC and KEM Hospital, Mumbai, India; Department of Nephrology, Fortis Hospital, Mumbai, India; Medgenome Labs Limited, Bangalore, India; Department of Nephrology, Seth GSMC and KEM Hospital, Mumbai, India; Department of Nephrology, Seth GSMC and KEM Hospital, Mumbai, India; Department of Nephrology, Seth GSMC and KEM Hospital, Mumbai, India; Department of Nephrology, Seth GSMC and KEM Hospital, Mumbai, India; Department of Nephrology, Seth GSMC and KEM Hospital, Mumbai, India; Department of Nephrology, Seth GSMC and KEM Hospital, Mumbai, India; Department of Nephrology, Seth GSMC and KEM Hospital, Mumbai, India; Department of Nephrology, Seth GSMC and KEM Hospital, Mumbai, India; Department of Nephrology, Seth GSMC and KEM Hospital, Mumbai, India; Department of Nephrology, Seth GSMC and KEM Hospital, Mumbai, India; Department of Nephrology, Seth GSMC and KEM Hospital, Mumbai, India; Department of Haematogenetics, ICMR-National Institute of Immunohaematology, KEM Hospital campus, Mumbai, India; Department of Haematogenetics, ICMR-National Institute of Immunohaematology, KEM Hospital campus, Mumbai, India; Department of Dentistry, Seth GSMC and KEM Hospital, Mumbai, India; Department of Dentistry, Seth GSMC and KEM Hospital, Mumbai, India; Department of Preventive and Social Medicine, Seth GSMC and KEM Hospital, Mumbai, India; Department of Endocrinology, Seth GSMC and KEM Hospital, Mumbai, India; Division of Nephrology, Jupiter Hospital, Thane, India; Department of Nephrology, Seth GSMC and KEM Hospital, Mumbai, India

**Keywords:** distal renal tubule, genetics, genotype–phenotype, rare kidney disease, renal tubular acidosis

## Abstract

**Background and hypothesis:**

Primary distal renal tubular acidosis (dRTA) is a rare inherited renal tubular disorder having a significant impact on growth and kidney function. Data on molecular genetics and long-term outcomes of primary dRTA, especially for newer genotypes, are limited.

**Methods:**

63 probands with a clinical diagnosis of dRTA underwent molecular genetic testing, specifically including *SLC4A1, ATP6V1B1, ATP6V0A4, WDR72*, and *FOXI1*. Genotype–phenotype characteristics and long-term outcomes were studied in this observational cohort study.

**Results:**

Diagnostic yield of genetic testing was 58.7%. Genotype positivity was associated with severe clinical and biochemical disease. *SLC4A1* (38.5%) was the most common genotype, followed by *WDR72* (13.5%) and *ATP6V1B1* (11.5%). *SLC4A1*: p.Ala858Asp (32.7%) was the exclusive biallelic variant detected (likely founder variant in the region). Five (9.6%) had variants of unknown significance. Notable features at initial presentation were delayed diagnosis (median 17 months), frequent failure to thrive [44 (78.6%)], rickets [40 (71.4%)], and hypokalaemic paralysis [11 (19.6%)]. Mean [(standard deviation (SD)] follow-up duration was 14.8 (11.7) years. 30 (53.6%) were >18 years of age at last clinical visit. Long-term follow-up was characterized by poor final height [mean (SD score): −3.0 (2.2)], persistent bone deformities [19 (33.9%)], and decreased eGFR [mean (SD): 89.4 (26.1) ml/min/1.73 m^2^]. The biallelic *SLC4A1*: p.Ala858Asp variant was characteristically associated with increased osmotic fragility of red blood cells, manifesting as haemolytic anaemia. *ATP6V1B1, ATP6V0A4*, and *FOXI1* variants were associated with sensorineural hearing deficit. All probands with *WDR72* variants manifested amelogenesis imperfecta. We report 13 novel genetic variants in primary dRTA and the fourth proband with a rare *FOXI1* variant.

**Conclusions:**

Primary dRTA in Asian Indians is a genetically diverse disease, and is characterized by delayed diagnosis, severe growth failure, bone deformities, and decreased kidney function in the long term. Findings from this study highlight the regional diversity and expand genotype–phenotype correlations in primary dRTA.

## Graphical Abstract

**Graphical Abstract:**
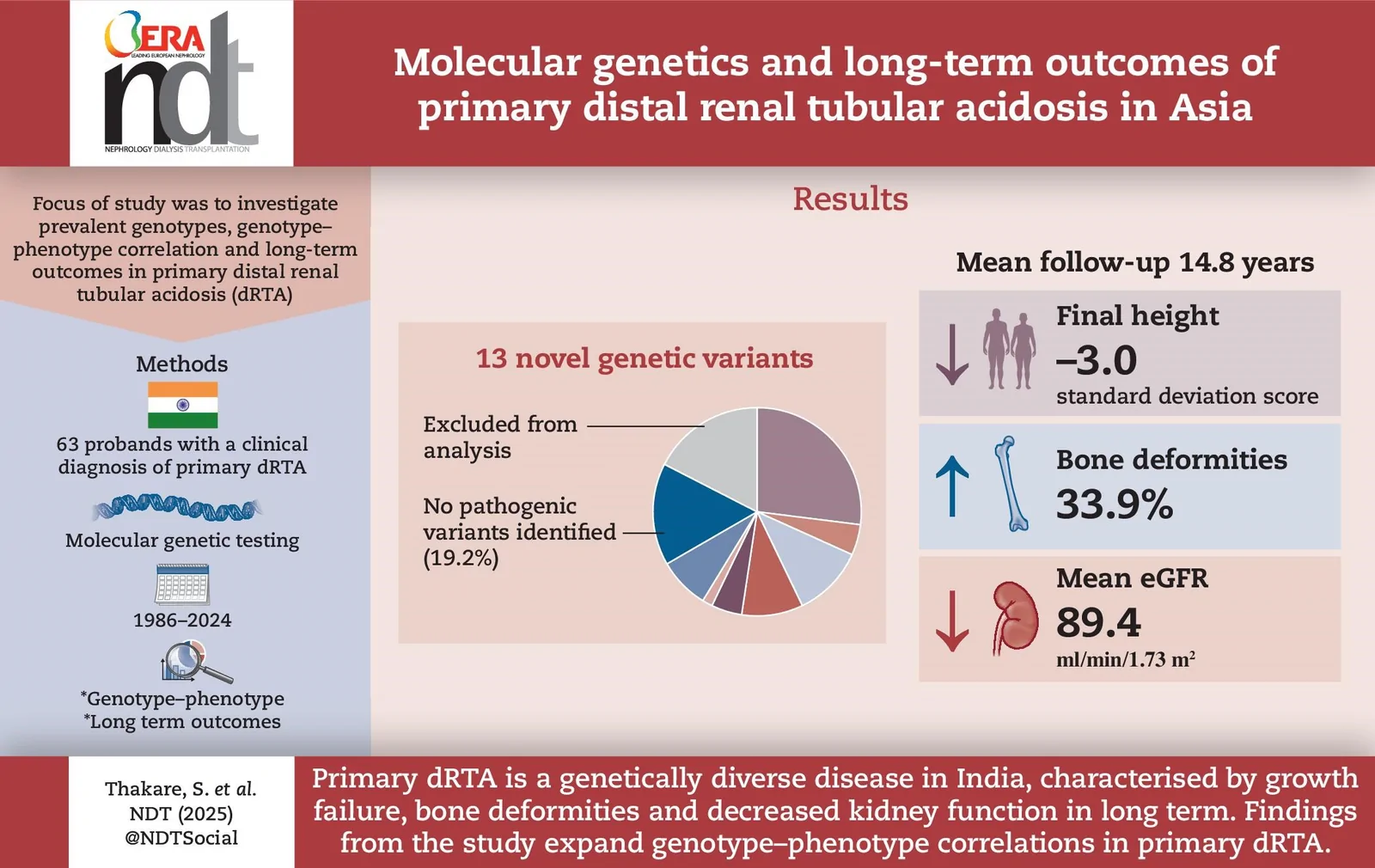

KEY LEARNING POINTS **What was known:**

- Underlying genetic defects in *SLC4A1, ATP6V1B1, ATP6V0A4*, and the novel genes *WDR72* and *FOXI1* account for 60%–80% of cases of primary distal renal tubular acidosis (dRTA).
- The knowledge of prevalence, clinical characteristics, and genotype–phenotype correlations in primary dRTA is still evolving, with significant geographical heterogeneity in the reported literature.
- Studies evaluating long-term impact of dRTA on renal and overall health are limited.

**This study adds:**

- *SLC4A1* is the most frequent genotype associated with dRTA in India. Biallelic *SLC4A1*: p.Ala858Asp is a likely founder variant in this region. It manifests a severe phenotype and is characterized by increased osmotic fragility of red blood cells in all patients, leading to a clinically variable haemolytic anaemia.
- *WDR72* is the second most frequent genotype in our region, having amelogenesis imperfecta as a characteristic feature. We report novel variants in *WDR72*, and highlight the phenotypic variability with this genotype. Our study also reports the fourth proband harbouring a novel *FOXI1* variant.
- Primary dRTA in our region carries a high burden of complications, notably, poor growth, persistent bony abnormalities and decreased kidney function in the long term.

**Potential impact:**

- This study expands the current knowledge of genotype distribution, phenotype, and long-term outcomes in primary dRTA. Clinical observations from this large cohort of patients with primary dRTA have immediate relevance to the clinical practice in this region.
- High burden of complications emphasizes the need for close follow-up and strict compliance to alkali therapy in primary dRTA, and underscores the importance of early diagnosis in modifying adverse long-term effects on growth and kidney function.
- This study highlights the genetic diversity of primary dRTA in India and adds to the growing body of evidence on genetic kidney diseases in the region. Comprehensive genetic testing is a valuable tool in the evaluation of overlapping phenotypes.

## INTRODUCTION

Primary distal renal tubular acidosis (dRTA) is a rare inherited renal tubular disorder. It is characterized by dysfunction of type-A intercalated cells in the late distal tubules and collecting ducts of the nephron, leading to defective urinary acidification. Primary dRTA presents in childhood with failure to thrive (FTT), renal (polyuria, nephrocalcinosis, nephrolithiasis), and/or bone manifestations (rickets). Genetic defects in *SLC4A1, ATP6V1B1*, and *ATP6V0A4* are known to account for 60%–80% of primary dRTA. Regional differences, with a dominance of *ATP6V1B1*/*ATP6V0A4* variants in Europe, and *SLC4A1* variants in Southeast Asia, have been observed [1–4]. Genetic diagnosis has evolved from a single-gene approach to whole-exome analysis using next-generation sequencing (NGS), leading to the identification of more recently described molecular defects associated with dRTA [5–7]. Specific genotypes are associated with characteristic extra-renal features such as haemolytic anaemia (*SLC4A1*), deafness (*ATP6V1B1, ATP6V0A4*, and *FOXI1*), and enamel defects (*WDR72*). Misdiagnosis or delayed diagnosis often leads to inadequate treatment, significantly affects the quality of life, and has an adverse impact on growth and long-term kidney function. Due to the rarity of this condition, insights on molecular genetics, clinical features, and disease progression are limited to a few large cohorts and case reports/series. This study was undertaken at a tertiary care referral centre in India to investigate the prevalent genotypes, genotype–phenotype correlations, and long-term outcomes in primary dRTA.

## MATERIALS AND METHODS

This study was conducted at the Renal Tubulopathy Clinic of Department of Nephrology, King Edward Memorial VII Hospital, Mumbai (Institutional Ethics Committee, EC/OA-28/2020). Patients with a diagnosis of dRTA characterized by persistent metabolic acidosis (serum bicarbonate <22 meq/l), normal anion gap (8–12 meq/l), hypokalaemia (serum potassium <3.5 meq/l) and defective renal acidification, evident by elevated urinary pH (>5.3), and/or positive urinary anion gap, were included. Secondary aetiologies (autoimmunity, obstructive uropathy, drugs/toxins) were excluded. Demographic, clinical, laboratory, and radiological features were noted from medical records (1986–2024). Clinical findings including anthropometry, skeletal deformities, hearing defects, and dental abnormalities were noted. Data from outpatient visits (3–6 monthly) were obtained from archived clinic records and patients’ files. Blood investigations included creatinine, electrolytes, bicarbonate, calcium, phosphorus, and haemoglobin. The estimated glomerular filtration rate (eGFR) was calculated using CKD-EPI formula (2019) in adults and the modified Schwartz formula for <18 years of age. Urinalysis included urine pH, electrolytes, citrate, and calcium creatinine ratio (UCCR). Serial renal ultrasounds were reviewed for findings of medullary nephrocalcinosis (MNC), nephrolithiasis (NL), and renal cysts. Details of treatment (alkali, potassium, thiazides, and other drugs) were noted. Alkali doses were titrated to achieve serum bicarbonate >22 meq/L and serum potassium >3.5 meq/L. Thiazides were used if hypercalciuria persisted after correction of acidosis. Results of dental evaluation, pure tone audiometry (PTA), brain stem evoked response audiometry (BERA), and eosin 5′ maleimide (EMA) dye-binding assay were noted.

Genetic testing was done for all probands. Genomic DNA was isolated from peripheral blood using standard techniques (Supplementary File). Molecular screening for >6000 genes (including *SLC4A1, ATP6V1B1, ATP6V0A4, WDR72*, and *FOXI1* associated with primary dRTA) was performed by NGS (Illumina) with >80–100 times depth of coverage. The functional implications of variants were predicted using *in silico* tools, and minor allele frequency was checked in TopMed and gnomAD databases. Variants were classified according to the American College of Medical Genetics and Genomics guidelines. STREGA guidelines were used for reporting the study.

### Statistical analysis

Statistical analysis was performed using IBM-SPSS software, version 26. Mean/standard deviation (SD) or median/interquartile range (IQR) were used to present normally and non-normally distributed data, respectively. Independent samples *t-*tests and Fisher’s Exact tests were used to compare quantitative and qualitative variables between two groups, respectively. Mann–Whitney *U*-test was performed to compare medians across two groups. Two-sided *P* values were reported. Missing variables were not imputed.

## RESULTS

**Figure 1:**
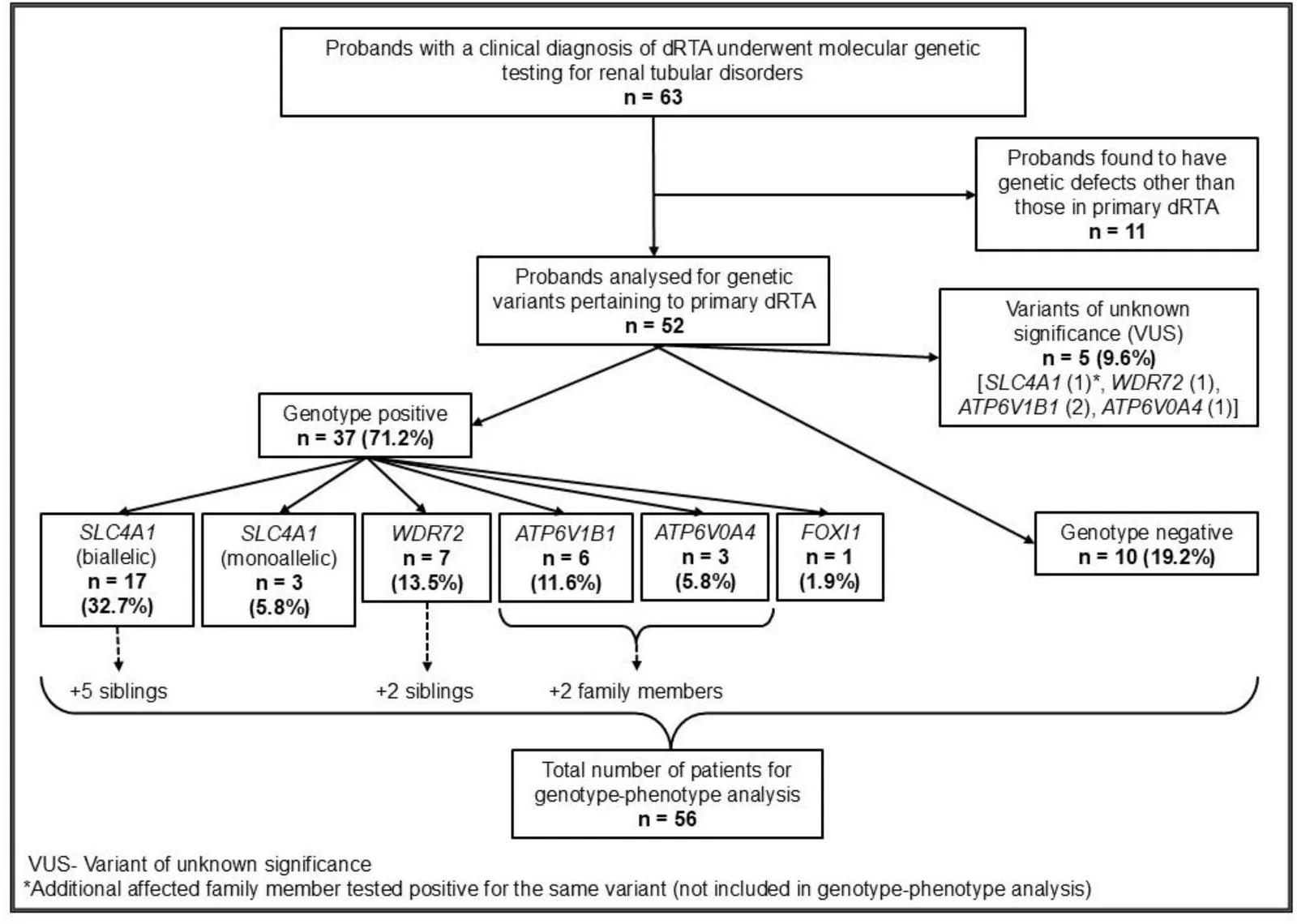
Study flow diagram showing distribution of genotypes in the cohort.

Molecular genetic testing was performed on 63 probands. Eleven probands, in whom genotyping was suggestive of other renal tubular disorders having a clinical and biochemical overlap with dRTA, were excluded. The remaining 47 probands, excluding five probands harbouring variants of unknown significance (VUS), were included in genotype–phenotype analysis (Fig. 1).

### Phenotype

#### Baseline characteristics at initial presentation

Phenotypic data from 47 probands and nine family members [*n* = 56, 29 females (51.8%)] are presented in Table 1. Parental consanguinity was noted in 19/47 (40.4%). Median age (IQR) at first clinical symptom, age at diagnosis, and delay in diagnosis were 12 (4–36) months, 45 (22.5–90) months, and 17 (1.5–42) months, respectively. At initial presentation, FTT, polyuria, rickets, and hypokalaemic paralysis (HKP) were present in 44 (78.6%), 39 (69.6%), 40 (71.4%), and 11 (19.6%) patients, respectively. Mean (SD) height SD score (SDS) at presentation was −3.9 (3.1), and short stature (height SDS <2 SDS) was noted in 29/36 (80.6%). Mean (SD) serum bicarbonate and urine pH at diagnosis were 12.8 (4.4) meq/L and 7.1 (0.7), respectively. Hypokalaemia, elevated UCCR (>0.2 mg/mg), hypocitraturia, and hypophosphatemia were seen in 46/53 (86.8%), 25/35 (71.4%), 33/36 (91.7%), and 30/47 (63.8%), respectively. Mean (SD) eGFR was 66.2 (23.7) ml/min/1.73 m^2^, and 15/40 (37.5%) had eGFR <60 ml/min/1.73 m^2^. Ultrasonographic evidence of MNC and/or NL was seen in 34/48 (70.8%).

**Table 1:** Baseline phenotypic characteristics of patients with primary distal renal tubular acidosis.

	Overall (*n* = 56; 47 probands)	*SLC4A1* (biallelic, *n* = 22; 17 probands)	*SLC4A1* (monoallelic, *n* = 3 probands)	*WDR72* (*n* = 9; 7 probands)	*ATP6V1B1* (*n* = 7; *n* = 6 probands)	*ATP6V0A4* (*n* = 4; 3 probands)	*FOXI1* (*n* = 1 proband)	Genotype-negative (*n* = 10 probands)
Age at first clinical symptoms (months): median (IQR)	12 (4–36)	12 (6–24)	18 (12–60)	60 (36–72)	3 (2.5–6)	3 (2.5–3)	0	96 (9–312)
Age at diagnosis (months): median (IQR)	45 (22.5–90)	48 (24–84)	60 (24–144)	84 (48- 168)	10 (3–36)	3.5 (3–8)	42	126 (24–348)
Delay in diagnosis (months): median (IQR)	17 (1.5–42)	28.5 (15–48)	12 (0–126)	27 (12–72)	3 (0–18)	1 (0.5–5)	42	13.5 (9–36)
Females (%)	29 (51.8)	11 (50)	2 (66.7)	8 (88.9)	4 (57.1)	1 (25)	1	2 (20)
Consanguinity (%) (probands only)	19/47 (40.4)	9/17 (52.9)	0/3 (0)	3/7 (42.9)	3/6 (50)	2/3 (33.3)	None	2/10 (20)
Family history of dRTA (%) (probands only)	8/47 (17)	4/17 (23.5) (One proband had two affected siblings)	0/3 (0)	2/7 (28.6)	1/6 (16.7)	1/3 (33.3)	0	0/10 (0)
FTT (%)	44 (78.6)	21 (95.5)	3 (100)	6 (66.7)	6 (85.7)	4 (100)	1	3 (30)
Polyuria (%)	39 (69.6)	18 (81.8)	3 (100)	7 (77.8)	5 (71.4)	3/4 (75)	1	2/10 (20)
HKP as presentation (%)	11 (19.6)	1 (4.6)	0 (0)	3 (33.3)	2 (28.6)	0 (0)	0	5 (50)
Rickets (%)	40 (71.4)	18 (81.8)	3 (100)	7 (77.8)	5 (71.4)	3 (75)	1	3 (30)
Height SDS: mean (SD) (*n* = 35)	−3.9 (3.1)	−4.4 (2.1)	−4.7 (2.1)	−3.6 (1.8)	−5.5 (3.3)	NA	−2.7	−2.2 (5.4)
Serum bicarbonate (meq/l): mean (SD) (*n* = 55)	12.8 (4.4)	10.6 (4.1)	10 (1.8)	13.2 (2.2)	13.1 (3.4)	13 (5.9)	9	17.7 (3.4)
Urinary pH: mean (SD) (*n* = 46)	7.1 (0.7)	7.2 (0.6)	7.3 (0.6)	7.7 (0.5)	6.6 (0.8)	6.7 (0.2)	7.00	6.7 (0.6)
eGFR (ml/min/1.73 m^2^): mean (SD) (*n* = 40)	66.2 (23.7)	67.3 (28.2)	70.2 (9.5)	73.1 (9.5)	49.4 (15)	NA	36.3	67.2 (30.6)
Hypokalaemia (%)	46/53 (86.8)	18/20 (90)	2 (66.7)	9 (100)	7 (100)	2 (50)	1	7/9 (77.8)
UCCR >0.2 mg/mg (%)	25/35 (71.4)	6/9 (66.7)	2 (66.7)	5 (55.6)	5/6 (83.3)	2 (66.7)	1	4/4 (100)
Hypophosphatemia (%)	30/47 (63.8)	10/17 (58.8)	2 (66.7)	7 (77.8)	4 (57.1)	1/1 (100)	1	5/9 (55.6)
MNC/NL (%)	34/48 (70.8)	14/19 (73.7)	3 (100)	5 (55.6)	4/5 (80)	3 (75)	0	5/7 (071.4)
Anaemia (Hb < 12 g/dl): mean (SD)	27/47 (57.5%)	15/16 (93.8%)	1 (33.3%)	2/8 (25%)	3/6 (50%)	1/3 (33.3%)	0	5/10 (50%)

#### Follow-up characteristics

**Figure 2:**
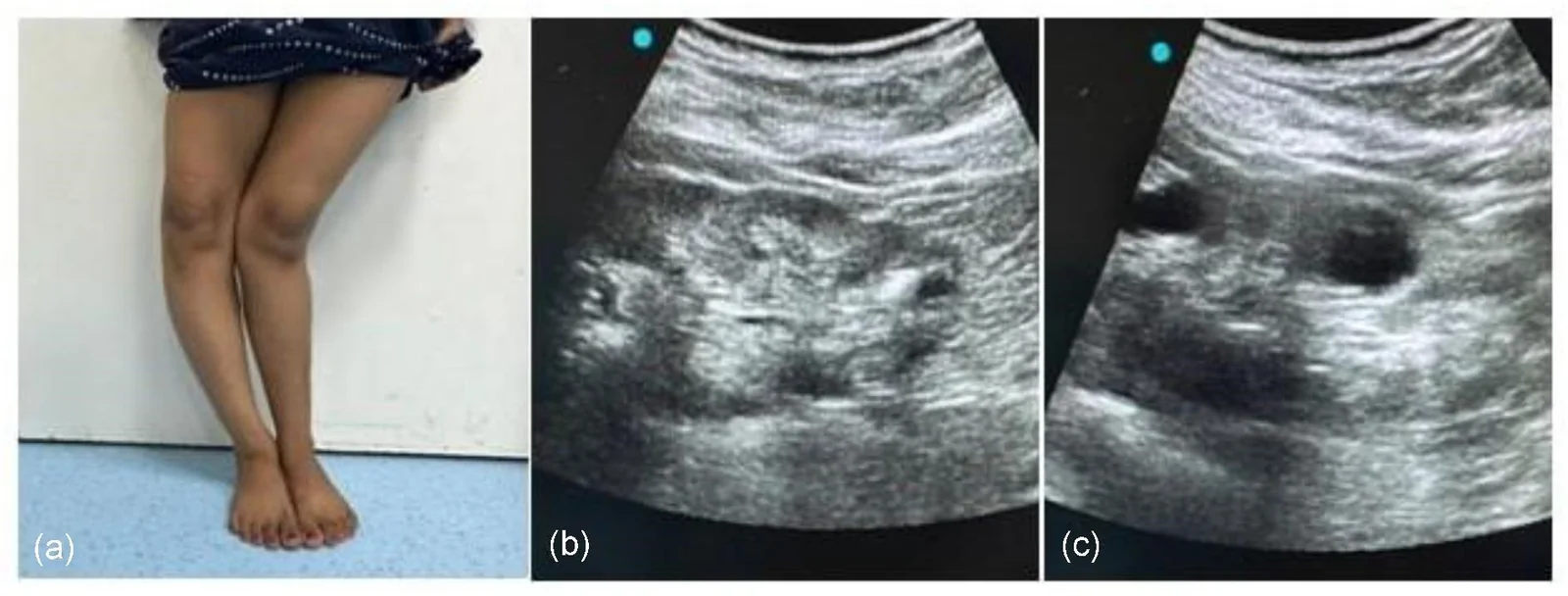
Long-term effects on growth and kidney function in distal renal tubular acidosis. (**a**) Persisting windswept deformity in a 25-year-old female proband with a *WDR72* variant. (**b**) Medullary nephrocalcinosis and (**c**) renal cysts in a 53-year-old male proband with a biallelic *SLC4A1*: p.Ala858Asp variant.

Table 2 shows the follow-up data of 47 probands and nine family members. Mean (SD) duration of follow-up and patient-age at latest follow-up were 14.8 (11.7) and 21.4 (13.3) years, respectively. Of them, 30 (53.6%) were adults at the last follow-up; 16 (28.6%) had the first HKP episode on further follow-up; 18 (32.1%) had multiple HKP episodes. Bone fracture(s) (receiving orthopaedic intervention) were noted in 17 (30.4%). Mean (SD) height SDS of the cohort was −3.0 (2.2), and bone deformities were present in 19 (33.9%). Five patients with bone deformities underwent surgical corrections. Mean (SD) doses of bicarbonate and potassium supplemented at the last follow-up were 3.2 (3.8) and 2.2 (2.7) meq/kg/day, respectively. Seventeen patients (30.4%) were treated with hydrochlorothiazide for hypercalciuria. Eight (14.3%) were additionally treated with spironolactone/enalapril for hypokalaemia/proteinuria. At the last follow-up visit, serum bicarbonate levels were >22 meq/l in 21/53 (39.6%), and UCCR was <0.2 mg/mg in 45/51 (88.2%) patients. Mean (SD) eGFR at the last follow-up was 89.4 (26.1) ml/min/1.73 m^2^. In total, 31 (55.4%) and seven (12.5%) patients had eGFR <90 and <60 ml/min/1.73 m^2^, and 12 (21.4%) patients had proteinuria. None of the patients had eGFR <15 ml/min/1.73 m^2^. MNC and/or NL were observed in 50 (89.3%) of the cohort, and 10 (17.9%) had renal cysts (Fig. 2). Five out of 56 (8.9%) patients developed episodes of erythrocytosis (haemoglobin >17 g/dl); one patient received a session of phlebotomy. Outcomes of seven gestations in five female probands were noted; four were uneventful. Spontaneous abortions (SA) were noted in two probands (one each with *ATP6V1B1* and *ATP6V0A4* genotype, the former experiencing recurrent SAs).

**Table 2:** Follow-up phenotypic characteristics of patients with primary distal renal tubular acidosis.

	Overall (*n* = 56; 47 probands)	*SLC4A1* (biallelic*, n* = 22; 17 probands)	*SLC4A1* (monoallelic*, n* = 3 probands)	*WDR72* (*n* = 9; 7 probands)	*ATP6V1B1* (*n* = 7; *n* = 6 probands)	*ATP6V0A4* (*n* = 4; 3 probands)	*FOXI1* (*n* = 1 proband)	Genotype-negative (*n* = 10 probands)
Age at last follow-up: mean (SD) years	21.4 (13.3)	19.8 (13.4)	21.3 (5.5)	24.1 (12.3)	21.4 (15.7)	16.8 (10.7)	35	23.2 (16.6)
Final height SDS: mean (SD)	−3.0 (2.2)	−3.4 (1.8)	−3.8 (1.7)	−3.4(2.4)	−2.2 (1.2)	−3.4 (1.4)	−2.3	−2.2 (3.7)
Bone deformities (%)	19 (33.9)	9 (40.9)	2 (66.7)	4 (44.4)	2 (28.6)	1 (25)	0	1 (10)
First episode of HKP occurring on follow-up (%)	16 (28.6)	9 (40.9)	0 (0)	3 (33.3)	2 (28.6)	2 (50)	0	0 (0)
Recurrent HKP (%)	18 (32.1)	4 (18.2)	0 (0)	5 (55.6)	3 (42.9)	2 (50)	0	4 (40)
Bicarbonate supplementation (meq/kg/d): mean (SD) (*n* = 55)	3.2 (3.8)	4.4 (5.2)	3.1 (2.2)	1.8 (1.4)	2.7 (1.8)	5.3 (3.5)	1.6	1.4 (1.2)
Potassium supplementation (meq/kg/d): mean (SD) (*n* = 48)	2.2 (2.7)	2.2(1.9)	1.8 (1.9)	1.5 (1.0)	3.8 (6.1)	2.6 (1.4)	0	1 (0.7)
eGFR (ml/min/1.73 m^2^): mean (SD)	89.4 (26.1)	91.7 (23.9)	87.3 (15.9)	103 (33.5)	86.9 (21.8)	80.6 (27.9)	91.6	77.7 (28.4)
eGFR <90 ml/min/1.73 m^2^ (%)	31 (55.4)	11 (50)	2 (66.7)	3 (33.3)	5 (71.4)	3 (75)	No	7 (70)
eGFR <60 ml/min/1.73 m^2^ (%)	7 (12.5)	2 (9.1)	0 (0)	1 (11.1)	0 (0)	1 (25)	No	3 (30)
Proteinuria (%)	12 (21.4)	4 (18.2)	1 (33.3)	2 (22.2)	2 (28.6)	1 (25)	No	2 (20)
MNC (%)	40 (71.42)	16 (72.7)	3 (100)	8 (88.9)	6 (85.7)	4 (100)	1	2 (20)
NL (%)	19 (33.9)	7 (31.8)	2 (66.7)	4 (44.4)	0	0	0	6 (60)
Either MNC/NL (%)	50 (89.3)	20 (90.9)	3 (100)	9 (100)	6 (85.7)	4 (100)	1	7 (70)

### Genotype

Out of 52 probands, 37 (71.2%) showed genetic variants classically described with primary dRTA (Table 3), with *SLC4A1* (20, 38.5%) being the commonest, followed by *WDR72* (seven, 13.5%), *ATP6V1B1* (six, 11.5%), *ATP6V0A4* (three, 5.8%), and *FOXI1* (one, 1.9%). None showed pathogenic variants in *ATP6V1C2. SLC4A1* (*n* = 20 probands): 17 had biallelic p.Ala858Asp variant; three had monoallelic variants (p.Arg589Cys in two, p.Arg589His in one). With *WDR72* (*n* = 7 probands), six had biallelic variants [p.Gly255ValfsTer40 in two, and p.Met100ArgfsTer22, p.Trp187Ter, p.Trp978Ter, chr15: g.(53616244_53665571)_(53665769_53699749)del in one each]; one had variants in compound heterozygous state [p.Ser388Ter and c.(1765+1_1766–1)_(1962+1_1963–1)del]. With *ATP6V1B1* (*n* = 6 probands), five had biallelic variants (p.Ile386HisfsTer56, p.Arg114Ter, p.Gln150SerfsTer14, p.Val125GlyfsTer10, and p.Asp6AlafsTer13); one had a pathogenic heterozygous variant (p.Arg394Gln). *ATP6V0A4* (*n* = 3 probands): two had homozygous variants (IVS15+1G>A and p.Arg807Gln); one had variants in compound heterozygous state (9IVS10+1G>A along with p.Arg389Trp). One proband had a missense variant (p.Arg213Leu) in the *FOXI1* gene. Of 26 variants overall, the above 21 were classified as pathogenic/likely pathogenic (novel, nine). Five were classified as VUS (previously reported, one; novel, four).

**Table 3:** Genetic variants observed in the study cohort.

Serial number	Gene	Variant	Zygosity	Mutation type	Protein change	Classification	Status (literature/database)
1.	*SLC4A1* NM_000342.4	Exon 19 c.2573C>A	Homozygous	Missense	p.Ala858Asp	Pathogenic (PS3, PM3, PM2, PP1, PP3)	Reported (PMID: 22919024, 37448902, 33068675)
2.		Exon 14 c.1765C>T	Heterozygous	Missense	p.Arg589Cys	Pathogenic (PS2, PM2, PM1, PP3)	Reported (PMID: 28233610, 32154456)
3.		Exon 14 c.1766G>A	Heterozygous	Missense	p.Arg589His	Pathogenic (PS2, PM2, PM1, PP3)	Reported (PMID: 28233610, 32154456)
4.		Exon 20 c.2716G>C	Heterozygous	Missense	p.Glu906Gln	VUS^(i)^ (PP3, PP4, BS2)	Reported (PMID: 25296721)
5.	*WDR72* ^a^ NM_182758.4	Exon 8 c.764_768del	Homozygous	Frameshift	p.Gly255ValfsTer40	Pathogenic (PVS1, PM2, PP1, PP4)	Reported (PMID: 31959358)
6.		Exon 3 c.299del	Homozygous	Frameshift	p.Met100ArgfsTer22	Likely pathogenic (PVS1, PM2, PP4)	Present study
7.		Exon 5 c.561G>A	Homozygous	Nonsense	p.Trp187Ter	Pathogenic (PVS1, PM2, PP5, PP4)	Reported (PMID: 910521)
8.		Exon 17 c.2934G>A	Homozygous	Nonsense	p.Trp978Ter	Pathogenic (PVS1, PM2, PP1, PP4)	Reported (PMID: 3910521)
9.		Exon 14 chr15:g.(53616244_53665571)_(53665769_53699749)del	Homozygous	CNV	c.(1765+1_1766–1)_(1962+1_1963–1)del	Likely pathogenic (2E, PP4)	Present study
10.		Exon 11 c.1163C>G	Compound Heterozygous	Nonsense	p.Ser388Ter	Likely pathogenic (PVS1, PM2, PP4)	Present study
		Exon 14 chr15:g.(53616244_53665571)_(5365769_53699749)del		CNV	c.(1765+1_1766–1)_(1962+1_1963–1)del	Likely pathogenic (2E, PP4)	Present study (same as in 10 above)
11.		Exon 7 c.743T>C	Homozygous	Missense	p.Leu248Pro	VUS^(ii)^ (PM2, PP4)	Present study
12.	*ATP6V1B1* NM_001692.4	Exon 12 c.1149dup	Homozygous	Frame shift	p.Ile386HisfsTer56	Pathogenic (PVS1, PM2, PP1, PP4)	Reported (PMID: 28233610, 31733597, 23923981, 28188436)
13.		Exon 4 c.340C>T	Homozygous	Nonsense	p.Arg114Ter	Pathogenic (PVS1, PM2, PP1, PP4)	Reported (PMID: 17669226, 28188436)
14.		Exon 6 c.448del	Homozygous	Frame shift	p.Gln150SerfsTer14	Likely pathogenic (PVS1, PM2, PP4)	Present study
15.		Exon 5 c.373dup	Homozygous	Frame shift	p.Val125GlyfsTer10	Likely pathogenic (PVS1, PM2, PP4)	Present study
16.		Exon 1 c.17_20del	Homozygous	Frame shift	p.Asp6AlafsTer13	Likely pathogenic (PVS1, PM2, PP4)	Present study
17.		Exon 12 c.1181G>A	Heterozygous	Missense	p.Arg394Gln	Likely pathogenic (PM2, PP3, PP1 strong, PP4)	Reported (PMID: 39837581, 26571219)
18.		Exon 10 c.1051C>T	Heterozygous	Missense	p.Pro351Ser	VUS^(iii)^ (PM2, PP3, PP4)	Present study
19.		Exon 5 c.377T>C	Heterozygous	Missense	p.Phe126Ser	VUS^(iv)^ (PM2, PP3, PP4)	Present study
20.	*ATP6V0A4* NM_020632.3	Intron 15 c.1572+1G>A	Homozygous	Splice site	IVS15+1G>A	Likely pathogenic (PVS1, PM2, PP4)	Present study
21.		Exon 3 c.2420G>A	Homozygous	Missense	p.Arg807Gln	Pathogenic (PS3, PM2, PM3, PP3, PP1, PP4)	Reported (PMID: 28233610, 12414817)
22.		Intron 10 c.816+1G>A	Compound heterozygous^b^	Splice site	IVS10+1G>A	Pathogenic (PVS1, PM2, PP4, PP5)	Reported (PMID: 28233610)
		Exon 12 c.1165C>T		Missense	p.Arg389Trp	Likely pathogenic (PM2, PM3, PP3, PP4)	Present study
23.		Intron 10 c.816+1G>A	Compound Heterozygous^c^	Splice site	IVS10+1G>A	Pathogenic (PVS1, PM2, PP4, PP5)	Reported (PMID: 28233610)
		Exon 21 c.2278A>C		Missense	p.Thr760Pro	VUS (PM2, PP3, PP4)	Present study
24.	*FOXI1* NM_012188.5	Exon 2 c.638G>T	Homozygous	Missense	p.Arg213Leu	Likely pathogenic (PM5, PM2, PP3, PP4)	Present study

^a^In our study, all *WDR72* variants were associated with both AI Type IIA3 and distal renal tubular acidosis (dRTA).

^b^Segregation of the variants was confirmed in parents;

^c^Mother was heterozygous for the c.816+1G>A variant; father’s sample was unavailable. In other probands with VUS, one or both parents were not available for testing (deceased).

For VUS: (i) The population frequency in TopMed and gnomAD is <0.0004. Damage by PolyPhen2 (HDiv/HVar) and MutationTaster2; (ii) Not present in TopMed and gnomAD. Damage by SIFT; (iii) Not present in TopMed and frequency of 0.00000657 in GnomAD, Damaging by PP2, SIFT, LRT, and MT2; and (iv) Not present in TopMed and GnomAD. Damage by PP2, SIFT, LRT, and MT2).

### Genotype–phenotype correlation

At baseline, genotype-positive probands (*n* = 37) had more severe disease than genotype-negative probands (*n* = 10), as evidenced by higher occurrence of FTT (94.6% vs. 30%, *P* = .000), polyuria (83.8% vs. 20%, *P* = .000), rickets (91.9% vs. 30%, *P* = .000), and lower serum bicarbonate (11.5 vs. 17.7 meq/l; *P* = .000) (Supplementary File). They were more likely to have MNC (83.8% vs. 20%; *P* = .001).

#### Biallelic defects

**Figure 3:**
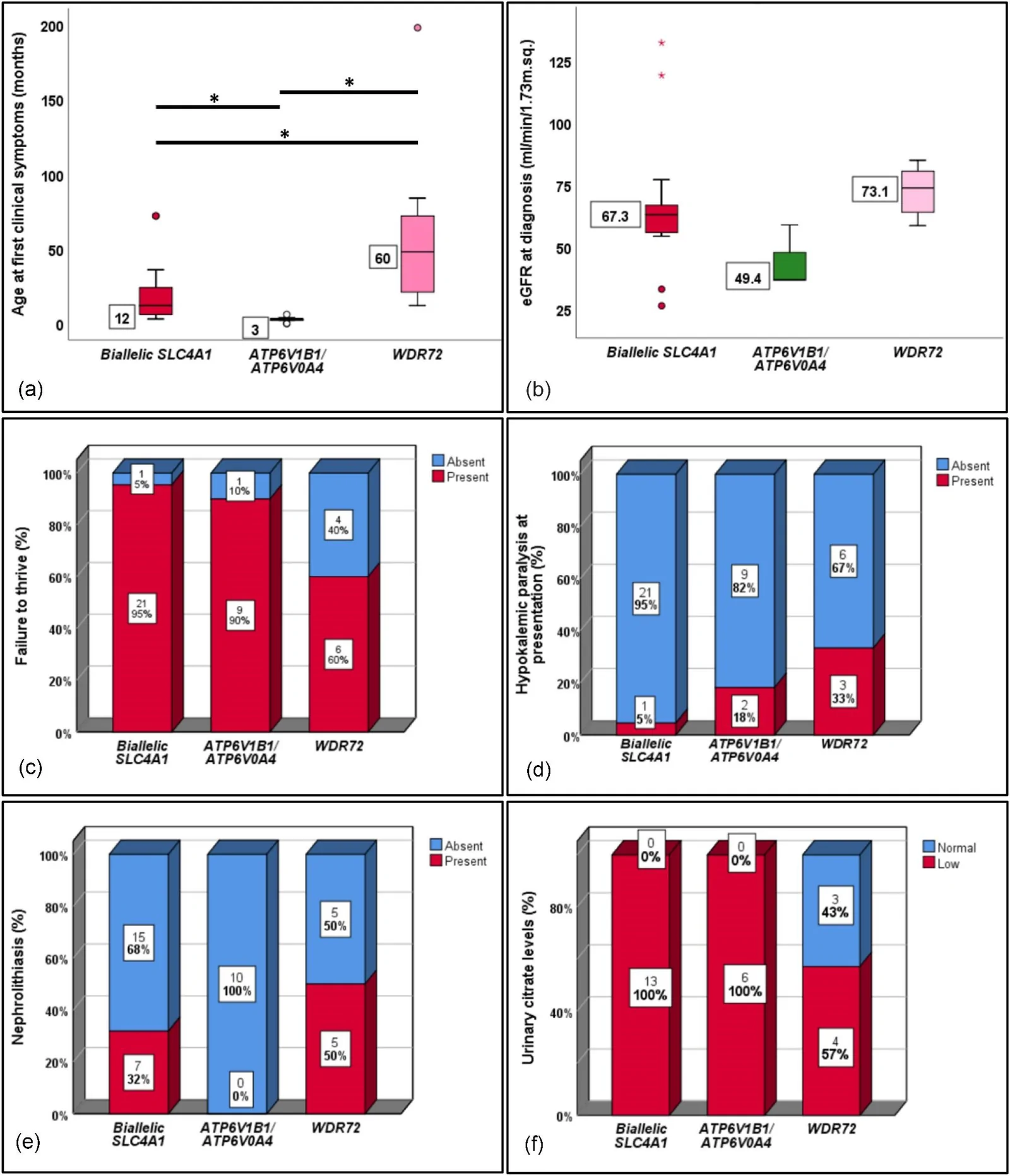
Key phenotypic differences between the genotype subgroups causing primary distal renal tubular acidosis- biallelic *SLC4A1, ATP6V1B1*/*ATP6V0A4*, and *WDR72*. (**a**)–(**f**) Median age at first clinical symptoms (**a**), mean estimated glomerular filtration rate (eGFR) at diagnosis (**b**), percentages of probands with FTT at presentation (**c**), hypokalaemic paralysis at presentation (**d**), nephrolithiasis (**e**), and urinary citrate levels (**f**). In (a) and (b), *(asterisk) indicates statistically significant differences between the subgroups (statistical analysis presented in the Supplementary File, Supplementary Table S3).

Salient phenotypic differences between biallelic genetic variants (*SLC4A1, ATP6V1B1/ATP6V0A4*, and *WDR72*) are depicted in Fig. 3. Ages at presentation of first clinical symptoms differed significantly (Supplementary File); the earliest in *ATP6V1B1/ATP6V0A4* [3 (2.5–4) months], followed by *SLC4A1* [12 (6–24) months] and *WDR72* [60 (36–72) months] variants. More patients with *SLC4A1* (95.5%) and *ATP6V1B1*/*ATP6V0A4* (90.9%) variants presented with FTT, compared with *WDR72* variants (66.7%). A history of sibling deaths due to FTT in infancy, was noted in four families (two each with *ATP6V1B1* and *ATP6V0A4* variants). Patients with *WDR72* variants often presented with an episode of HKP (33.3%) and tended to have recurrent episodes (66.7%). Two of these had normal urinary citrate excretion, and 44.4% had NL, in contrast to others.

#### Specific phenotypic features: haemolytic anaemia

**Figure 4:**
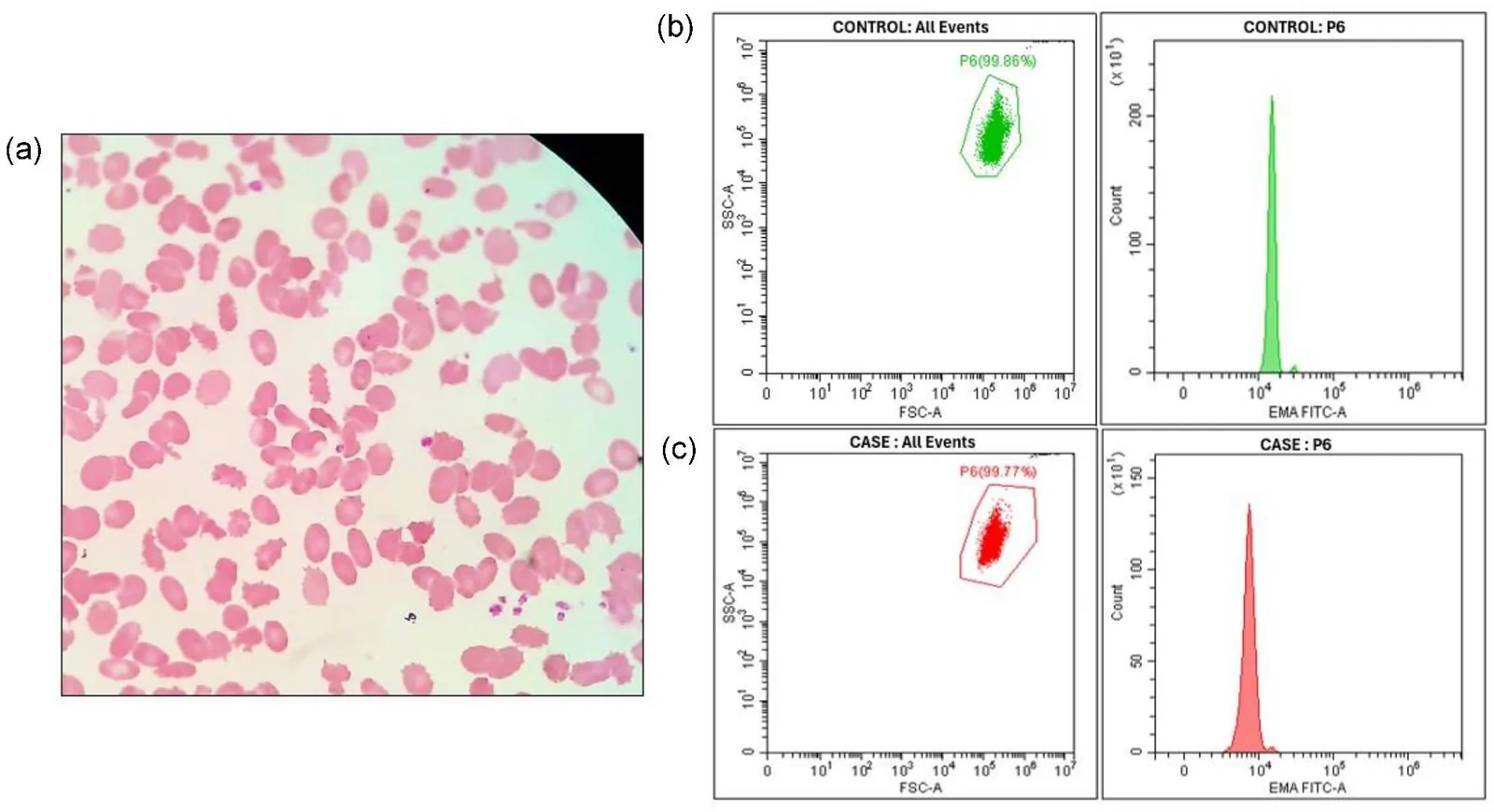
Haemolytic anaemia associated with biallelic *SLC4A1*: p.Ala858Asp variant. (**a**) Peripheral smear showing anisopoikilocytosis, spherocytes, and elliptocytes in a proband with biallelic *SLC4A1*: p.Ala858Asp. Eosin 5′ maleimide (EMA) binding test results of (**b**) normal control versus (**c**) a proband with biallelic *SLC4A1*: p.Ala858Asp. FSC-A (forward scatter) and SSC-A (side scatter) area plots are shown for each in the panels. The gate-shaded areas in green and red in the plots are the singlet RBCs. Data from the gate are plotted in the EMA histogram (right). FITC-A, fluorescein isothiocyanate-area.

Most patients with biallelic *SLC4A1*: pAla858Asp variant had anaemia at initial presentation (93.8% vs. 57.5% overall). Seven out of 22 (31.8%) received blood transfusion(s), or had episodes of jaundice [neonatal; *n* = 6 (27.3%), and later; *n* = 13 (59.1%)], or persistent jaundice (*n* = 2, symptomatic cholelithiasis in one). Evidence of red cell membrane defect was documented in 15/15 (100%) patients with biallelic *SLC4A1*: pAla858Asp (Supplementary File), where EMA dye-binding assay uniformly showed mean channel fluorescence <65 000 units (Fig. 4).

#### Specific phenotypic features: sensorineural hearing deficit (SNHD)

Audiometry (PTA/BERA) was performed in 13/14 patients with *ATP6V1B1/ATP6V0A4* variants. SNHD was detected in seven patients (four with *ATP6V1B1*, three with *ATP6V0A4*). SNHD in six patients manifested before 10 years of age (supplementary file). Notably, SNHD at higher frequencies was noted in two probands; one each with heterozygous *ATP6V1B1*: p.Phe126Ser and *WDR72*: p.Gly255ValfsTer40, at ages 49 years and 7 years, respectively.

#### Specific phenotypic features: amelogenesis imperfecta (AI)

**Figure 5:**
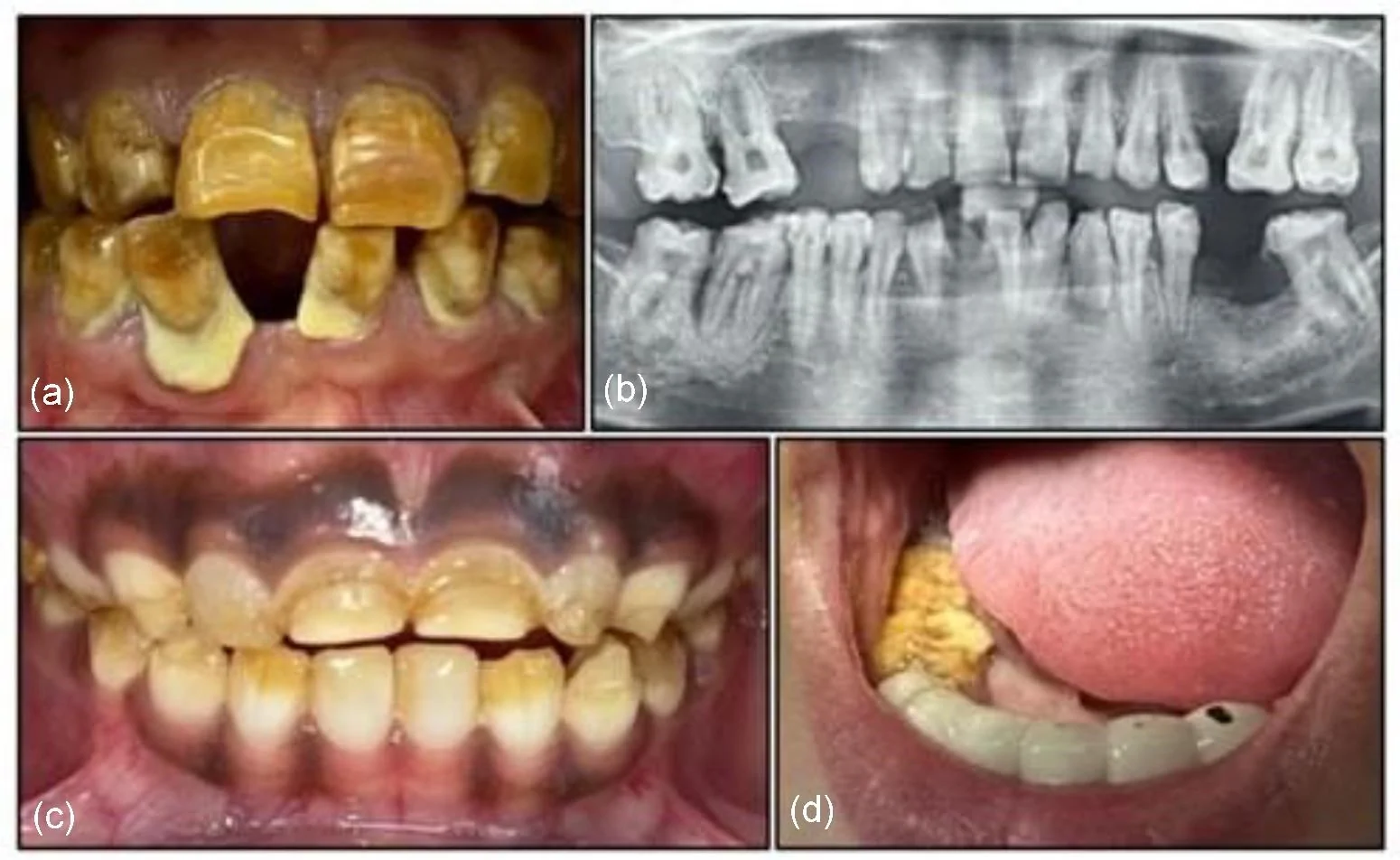
Gallery of dental findings in patients with *WDR72* variants. (**a**) Yellowish-brown discoloration of teeth, soft enamel with mottled, notched appearance in a proband with hypoplastic hypomaturation AI type IIA3. Heavy calculus deposits in all lower anterior teeth, causing displacement of teeth with an anterior open bite. (**b**) Orthopantomogram of the same proband showing thin-layered enamel with radiodensity more than dentin. (**c**) Yellowish discoloration of teeth observed in another proband. Enamel is completely chipped off from the incisal and occlusal surfaces of all teeth, except in mandibular central incisors, exposing the dentin. Periodontium shows generalized papillary gingivitis. (**d**) Premolar to premolar porcelain fused to metal crown for aesthetic correction.

All 10 (100%) patients manifested abnormalities in dental enamel consistent with AI type IIA3 (Fig. 5). The teeth were yellowish-brown, enamel was creamy to hard, and chipping was noted. All teeth were uniformly affected. Parents reported noticing yellowish-brown deciduous teeth in all patients. The maternal uncle of proband with *WDR72*: p.Met100ArgfsTer22 manifested AI, but had no biochemical evidence of dRTA. Notably, partial AI was also observed in two probands with biallelic *SLC4A1*: p.Ala858Asp (Supplementary File).

#### Proband with FOXI1 variant

A female proband with biallelic *FOXI1*: p.Arg213Leu was diagnosed with dRTA at 3.5 years of age. Parental consanguinity was absent. The patient was symptomatic with early-onset FTT, delayed motor milestones, and rickets; however, she never experienced HKP. Temporal bone imaging showed bilaterally enlarged vestibular aqueducts. SNHD and MNC were detected at 8 and 14 years, respectively. At 35 years of age, she is short-statured (height SDS −2.3), has no bone deformities, and has a biochemically well-controlled disease. eGFR at last follow-up was 91.6 ml/min/1.73 m^2^.

#### Monoallelic defects

Most patients had biallelic variants, except few with *SLC4A1* (three probands) and *ATP6V1B1* (proband and her father). Median age (IQR) at first clinical symptoms for monoallelic *SLC4A1* variants was 18 (12–60) months. Patients had a similar clinical and laboratory profile, in comparison to other recessive genotypes (Tables 1 and 2). In the *ATP6V1B1* group, a father–daughter pair had a heterozygous variant (p.Arg394Gln). The daughter was symptomatic in infancy (3 months) with FTT, MNC, and SNHD, whereas the father presented at 3 years of age with HKP, without FTT or rickets. He did not have SNHD until 45 years of age.

#### Probands with VUS (*n* = 5)

Five (9.6%) probands had VUS (*SLC4A1* one, *WDR72* one, *ATP6V1B1* two, and *ATP6V0A4* one compound heterozygous) (Table 3). A proband–sister pair, both harbouring heterozygous *SLC4A1*: p.Glu906Gln, presented in adulthood (25 and 34 years, respectively) with HKP, had normal stature without bone deformities, and late-onset MNC/NL. The proband with a missense VUS (p.Leu248Pro) in *WDR72* (41 years) had classical manifestations of dRTA and AI. Two probands with heterozygous *ATP6V1B1* (p.Pro351Ser and p.Phe126Ser) had a late presentation with HKP (7 and 29 years, respectively), and MNC/NL developed much later at follow-up (24 and 34 years, respectively). Proband with p.Phe126Ser was detected to have mild SNHD to higher frequencies at 49 years of age. These two patients with heterozygous *ATP6V1B1* variants required lower doses of bicarbonate (1.5 and 1.2 meq/kg/day) and potassium (0.7 and 1.2 meq/kg/day) compared with cohort means (Table 2). Parents were not available for testing in all probands with VUS, except the mother (age 54 years) of a proband with heterozygous *ATP6V1B1*: p.Pro351Ser. She harbours the same variant, but has no biochemical evidence of dRTA or SNHD.

#### Other genotypes (*n* = 11)

Eleven probands were found to have other genetic defects with a phenotype overlapping with dRTA: mixed RTA (*CA2, SLC7A7*), disorders with dRTA as an association (*UMOD, CYP21A2, MAGED2, AIRE, ATP7B*), and disorders mimicking dRTA at initial presentation [Bartter’s syndrome (*SLC12A1, KCNJ1*) and protein-losing enteropathy (*DGAT1*)] (Supplementary File).

## DISCUSSION

Notable features of primary dRTA from our cohort were delayed diagnosis and severe clinical manifestations such as FTT, rickets, and frequent HKP at initial presentation. Growth failure, persistent bone deformities, and decreased eGFR were observed in long-term. The commonest genotype was *SLC4A1*, followed by *WDR72* and *ATP6V1B1*. We report 13 novel genetic variants in primary dRTA and a rare *FOXI1* variant. This is the largest cohort of dRTA reporting genotype–phenotype correlations for all currently known genotypes implicated in primary dRTA. This is also one of the largest reported cohorts of *SLC4A1:* p.Ala858Asp and *WDR72* genotypes.

The yield of genetic testing was 58.7% in our cohort, varying from 64% to 88.3% in previous studies [2, 8–10]. Manifestations of FTT, polyuria, rickets, and lower serum bicarbonate at first presentation were predictors of genotype positivity. Delayed diagnosis and a higher incidence of FTT, rickets, and HKP were noted compared with others, indicating a severe initial clinical presentation in Asian dRTA [2, 8, 11, 12]. Early growth impairment, resulting from delayed diagnosis, may explain the poor final height in our cohort, compared with others [2, 8, 12]. Mean (SD) urinary pH at initial presentation in our cohort was 7.1 (0.6). ∼74% of patients had urine pH >7. None of the patients (except one genotype-negative proband) had urine pH <6. Our findings, and those from a previous report systematically reporting urinary pH (2), suggest that the cut-off for primary dRTA may be higher than previously defined (>5.3) [13].

At a mean follow-up of ∼15 years, none of the patients developed end-stage kidney disease, although a comparatively higher proportion (73.1% children and 40% adults) had eGFR <90 ml/min/1.73 m^2^ at last follow-up [2, 8, 12]. Of the patients, 7.7% children and 16.7% adults developed KDIGO CKD Stage 3 (eGFR <60 ml/min/1.73 m^2^). Only ∼40% had good metabolic control (serum bicarbonate >22 meq/l) at last follow-up, similar to another cohort [8].

Our findings, indicating *SLC4A1* as the commonest genotype followed by *WDR72*, add to the current understanding of regional diversity in primary dRTA. Geographical variation in this disease is remarkable. dRTA caused by biallelic *SLC4A1* variants (‘tropical dRTA’) is the most common form of dRTA in South Asia [4]. By contrast, *ATP6V1B1, ATP6V0A4* and monoallelic *SLC4A1* (rare in South Asia) are the predominant genotypes in European [2, 8], and other non-tropical countries [9, 14]. The exclusivity of biallelic *SLC4A1*: p.Ala858Asp in our cohort, coupled with previous case reports/series from India [10, 15–19], including one study demonstrating an invariable carrier state in both parents [17], suggests a high likelihood of this being a founder variant in our region. Notably, this *SLC4A1*: p.Ala858Asp variant is more commonly reported from southern (tropical) parts of India, whereas *ATP6V1B1*/*ATP6V0A4* and monoallelic *SLC4A1* variants appear to be common in northern (non-tropical) regions [10, 20, 21]. *WDR72* (13.5%) was the second most common genotype, suggesting a higher prevalence in our region [22].

Our data suggest that age at first clinical symptoms can provide a clue towards the underlying genetic defect in dRTA. In our cohort, biallelic *ATP6V1B1*/*ATP6V0A4* variants manifested early, invariably in the first quarter of infancy, whereas biallelic *SLC4A1* and *WDR72* variants manifested in early and late childhood, respectively. Also, another discriminatory feature is the nature of initial presentation. Probands with biallelic *SLC4A1* and *ATP6V1B1*/*ATP6V0A4* frequently presented with FTT, as opposed to probands with *WDR72*, who presented with musculoskeletal features (HKPs, fractures, deformities) rather than growth failure. Biallelic *SLC4A1*-dRTA can be said to have a severe phenotype, unlike monoallelic *SLC4A1*-dRTA [2], since clinical and laboratory features, notably alkali and potassium requirements, were similar to *ATP6V1B1/ATP6V0A4* variants.

The N-terminal domain of SLC4A1 protein (Band 3 or AE1), the most abundant RBC membrane protein, binds to cytoplasmic ankyrin and maintains RBC morphology, whereas the C-terminal domain is responsible for chloride/bicarbonate exchange [23]. Presence of an environmental factor (endemic malaria) possibly favoured local evolution of *SLC4A1* variants in this region. Cation leakiness (conferred by mutated AE1) is speculated to interrupt the life cycle of malaria in host RBCs [24–26]. Haemolytic anaemia (varying from mild to severe, episodic to persistent) was a common clinical feature in our study. EMA dye-binding assay has an excellent predictive value in identifying the characteristic RBC membrane defect. This osmotic fragility testing can be useful, especially when genetic testing is not feasible, and may obviate the need of extensive evaluation for anaemia [18].

Biallelic *ATP6V1B1/ATP6V0A4* variants caused an early-onset, severe form of disease, often presenting with an intercurrent illness precipitating severe dehydration and electrolyte imbalances. This also likely explains the lowest eGFRs at presentation in this group. Severe FTT may confer an increased susceptibility to infections. Despite the anticipated severity of disease with *ATP6V1B1/ATP6V0A4* genotypes, final height, and eGFR in these groups were comparable to others, suggesting benefits of early diagnosis and alkali therapy.

Notably, the heterozygous *ATP6V1B1*: p.Arg394Gln variant from our cohort is similar to that reported in a large European cohort, and several others [2, 27–29]. A recent *in silico* protein energetics analysis using the FoldX modelling suite, gives strong evidence for pathogenicity of this heterozygous variant in dRTA [30]. We present their detailed phenotype and follow-up, not available from previous reports [31–33]. We also observed a definitive dRTA phenotype in two other cases with heterozygous VUS in *ATP6V1B1* (p.Pro351Ser and p.Phe126Ser). We cannot exclude abnormal gene dosing [2, 34] or functional gene variants in regulatory regions in these cases.

*WDR72*-dRTA is being reported increasingly from South Asia [22, 35, 36]. The protein, WDR72, directs microtubular assembly, involved in vesicle transport and membrane mobilization, crucial for physiological enamel mineralization [37]. All cases in our study showed characteristic enamel defects. In kidneys, these variants possibly lead to intracellular retention or mistargeting of proteins (kAE1 and/or V-type-ATPase). Notably, high-frequency SNHD was observed in a 7-year-old proband with *WDR72* variant, similar to a previous report [38].

A remarkable feature of our *WDR72* group was phenotypic variability, reported previously as ‘rate-dependent’ dRTA, often manifesting with a variable metabolic acidosis and retained ability to acidify urine [36]. Variable clinical features, presence of normal urinary citrate in some, and comparatively lower requirement of alkali and potassium doses, appear to reflect this biochemical phenomenon. At least three patients reported being asymptomatic without therapy for prolonged periods. In addition, a family member of one proband manifested only AI without metabolic acidosis. Phenotypic variability may lead to delay in diagnosis and non-compliance to alkali therapy, increasing the risk of adverse impact on growth, skeletal health, and kidney function. Indeed, a significant proportion of patients with *WDR72*-dRTA experienced recurrent HKPs, bone deformities, and structural kidney changes [MNC, NL, cysts]. Nonsense-mediated loss of function is presumed to be the dominant mechanism of pathogenesis in *WDR72*-dRTA [37]. Phenotypic variability may be attributable to the class of variants encountered in our cases, significantly different from a recent large cohort [22]. Other modifiers in the genome may also contribute to this effect.

We report the fourth proband with a novel homozygous variation in exon 2 of *FOXI1* gene causing dRTA. *In silico* predictions of this variant (p.Arg213Leu) were probably damaging. A different missense variant (p.Arg213Pro) in the same codon was shown to reduce DNA-binding, leading to decreased production of target genes (B1 and A4 subunits of V-type ATPase, AE1, AE4, and pendrin) [6]. It is not surprising that *FOXI1*-dRTA phenotypically mimics dRTA caused by *ATP6V1B1*/*ATP6V0A4* variants, similar to our observations. Importantly, 11 probands were excluded from analysis at the outset, on discovery of genetic variants reported with other disorders; most of them were later confirmed by reverse phenotyping. However, initial presentation in these probands mimicked dRTA: mixed RTA two [39–41], disorders associated with dRTA five [42–46], Bartter’s syndrome three [47–49], and congenital diarrhoea one [50] Though metabolic acidosis was an unexpected initial finding in the last two scenarios, similar observations have been noted previously [47–50]. This highlights the clinical utility of NGS versus targeted-panel sequencing while dealing with overlapping phenotypes.

Notable strengths of this study are detailed phenotype characterization, longitudinal follow-up at a dedicated renal tubulopathy clinic, and the use of NGS. We report the longest recorded follow-up durations (mean ∼15 years), with several findings of immediate relevance to clinical practice in the region. Limitations include lack of further evaluation of VUS variants by familial segregation analysis or *in vivo*/*in vitro* testing for functional significance. Additional genetic testing (chromosomal microarray or whole genome assay) could not be performed for genotype-negative probands, and for screening the second allele in individuals with heterozygous *ATP6V1B1* variants. We hypothesize that AKI due to intercurrent illness in some patients could be a cause for lower initial eGFR values compared with the last follow-up. However, as most of our patients were followed up from infancy to adulthood, the change in the estimating equation (modified Schwartz to CKD-EPI) with age may also have influenced the observed eGFR values.

## CONCLUSIONS

Primary dRTA is a genetically diverse disease in Asian Indians, and carries a significant burden of renal and extra-renal complications. Findings from this study expand the current knowledge of genotype distribution, phenotype, and long-term clinical outcomes in primary dRTA.

## Supplementary Material

gfaf222_Supplemental_File

## Data Availability

Data supporting findings of this study can be shared upon request.
