# Supplementary material for: Molecular genetics and long-term outcomes of primary distal renal tubular acidosis in Asia
Source: Nephrol Dial Transplant. 2025 Oct 24;41(5):942–55. doi: 10.1093/ndt/gfaf222 (PMC13232045; doi:10.1093/ndt/gfaf222)
Supplement: gfaf222_Supplemental_File [file gfaf222_supplemental_file.pdf]

Thakare S, Lila A, Keskar V, et al. ‘Molecular genetics and long-term outcomes in Asian Indian patients with primary distal renal tubular acidosis: an emphasis on regional diversity’.

<u>Supplementary material</u>

Index:

1. Supplementary methods
   A. Next Generation Sequencing
   B. Eosin 5’ maleimide (EMA) dye-binding test
   C. Definitions used in the study
   D. eGFR estimation for the study
2. Supplementary table 1: Genotype of excluded probands harbouring variants in genes associated with other disorders
3. Supplementary table 2: Comparison between characteristics of genetic versus non-genetic forms dRTA
4. Supplementary table 3: Comparisons between recessively inherited dRTA groups (*SLC4A1*, *ATP6V1B1*/*ATP6V0A4* and *WDR72*)
5. Supplementary figure 1: Phenotypic characteristics: Partial Amelogenesis Imperfecta in two probands with *SLC4A1*: p.Ala858Asp mutation
6. Supplementary table 4: Phenotypic characteristics: Occurrence of sensorineural hearing deficit (SNHD) in overall cohort with age at onset
7. Supplementary Table 5: Results of Eosin 5’ Maleimide (EMA) dye-binding test for detecting red blood cell osmotic fragility

1. *Supplementary methods:*

A. Next Generation Sequencing (NGS):

a) Methodology: Genomic DNA was isolated from whole blood using QIAamp DNA Blood Mini Kit (Qiagen, Germany) and quantified using Qubit fluorometry (Thermo Fisher Scientific, USA). For library preparation, 200 ng of the Qubit quantified DNA was fragmented to ~350 bp inserts. The library was hybridised and enriched using ~16.5 Mb custom exome panel (Nimblegen, CA) consisting of >6000 genes, including the clinically important ~23 genes. Library was assessed for fragment size distribution using Tape Station (Agilent, USA) and was quantified using Qubit (Thermo Fisher Scientific, USA) and sequenced as 2 × 150 bp paired-end reads on an Illumina HiseqX/NovaseqX plus (Illumina, CA) machine according to the manufacture's protocol. The library was sequenced to an average sequencing depth of ≥80-100x.

b) Variant calling and annotation:

Following quality check and adapter trimming using fastq-mcf (version 1.04.676), the sequencing reads obtained are aligned to human reference genome (GRCh38.p13) using BWA program [PMID: 20080505; PMID: 23155063]. The aligned reads were sorted, and duplicate reads were removed and the variants were called using GATK best practices pipeline using Sentieon (v201808.07). Gene annotation of the variants was performed using VEP program [PMID: 27268795] against the Ensembl release 99 human gene model. The variants were annotated for allele frequency [population databases GnomAD (v3.0), 1000 genome, MedGenome population specific database], *in silico* prediction tools [CADD, PolyPhen-2, SIFT, Mutation Taster2, and LRT] and disease databases [OMIM (Feb 2020), ClinVar (Feb 2020) and HGMD (v2019.4)]. The quality of the variant was analysed using integrative genomics viewer (igv) [PMID: 21221095]. In addition to single nucleotide variants (SNVs) and small Indels, copy number variants (CNVs) are detected from targeted sequence data using the ExomeDepth (v1.1.10) method.

c) Pipeline to narrow down potential gene and variant selection:

The variants with a minor allele frequency of >5% in population databases were filtered out. The variations were further prioritised based on genotype-phenotype association using Human Phenotype Ontology (HPO) and OMIM phenotype. The filtered variations were assessed for conservation, *in silico* prediction effect, known association in literature and disease databases (HGMD, ClinVar, and OMIM). Pathogenicity of the prioritised variant was ascertained using American College of Medical Genetics and Genomics (ACMG) standards and recommendation (Richards et al., 2015).

B. Eosin 5′ maleimide (EMA) dye-binding test

An alternative and gold standard flow cytometric based methodology for the diagnosis of HS is the measurement of mean fluorescence intensity (MFI) units using eosin-5′-maleimide (EMA) dye for verifying the loss of surface area of RBCs. The EMA interacts covalently with Lys-430 in the first extracellular loop of band 3 protein via its maleimide moiety, where the eosin chromophore is supposed to be held in a pocket deep inside transmembrane core of band 3.

Eosin 5′ maleimide (0.5 mg/mL PBS; *Fluka*, Gillingham, UK) dye-binding assay is performed for suspected cases of haemolytic anaemia, followed by imaging flow cytometry (AmnisImageStream®X Mark II, Seattle, WA). EDTA blood samples (2.0 mL) are used. Five microliters of red blood cells are washed with phosphate buffered saline (PBS) and incubated for an hour with 25 µL of EMA dye (0.5 mg/mL in PBS, Fluka, Gillingham, UK) in dark at room temperature with intermittent mixing. RBCs after incubation are centrifuged at 11,000 RCF for 2 min. Supernatant with unbound dye is removed carefully. RBC pellets are then washed three times with PBS–bovine serum albumin (BSA) solution (0.5% BSA in PBS) for 2 min at 11,000 RCF. Washed cells are resuspended in 500 µL of PBS. One hundred microliters of labeled cells are resuspended in 1.4 mL of 0.5% PBS for flow cytometric analysis. The intensity of dye fluorescence is determined in the fluorescence channel-2 (FL-2) for 15,000 events on

the Amnis ImageStream®X Mark II flow cytometer. IFC 488 nm Blue laser (200 mW) with 40× magnification was used for detection of fluorescence dye and Side scatter (SSC) images were produced from a dedicated 785 nm laser with 2 mW of power. The results of flow cytometric analysis of EMA labelled red cells are expressed in mean fluorescence intensity (MFI) units.

C. Definitions used in the study:

- Delay in diagnosis- Time period between onset of symptoms suggestive of dRTA (failure to thrive, polyuria, hypokalaemic paralysis) and correct biochemical diagnosis of dRTA
- Failure to thrive (FTT)- Presenting complaint of a patient; comprising of features of insufficient weight gain and absence of appropriate physical growth in infancy or childhood
- Height Standard Deviation Scores (SDS) were calculated as per growth charts from Indian Academy of Pediatrics (IAP)

  Reference: Indian Academy of Pediatrics Growth Charts Committee, Khadilkar V, Yadav S, et al. Revised IAP growth charts for height, weight and body mass index for 5- to 18-year-old Indian children. *Indian Pediatr*. 2015;52(1):47-55. doi:10.1007/s13312-015-0566-5
- Proteinuria- More than two consecutive urine samples showing presence of urinary protein (24-hour urinary protein >300 mg/day or spot urinary protein >1+).
- Hypercalciuria- Urine calcium creatinine ratio > 0.2 mg/mg for all age groups
- Hypophosphatemia- Serum phosphorus < 2.5mg/dl

D. eGFR estimation for the study:

Serum creatinine was measured by Modified Jaffe's method in our study. eGFR was calculated using Modified Schwartz method for <18 years of age and by CKD-EPI formula for >18 years of age. Equations such as the FAS formula [1] estimates eGFR better across the full age spectrum, however requires computation of Q-values to normalise serum creatinine concentrations within the same age and sex groups. As pointed out by Bisen et al [2], Q-values are likely to be specific for specific populations. It is unclear whether Q-values will be

applicable to all ethnicities. Median serum creatinine values from our population based on age and sex have not been systematically studied, hence calculation of eGFR by this method was not possible for our study.

Reference 1: Pottel H, Hoste L, Dubourg L, et al. An estimated glomerular filtration rate equation for the full age spectrum. *Nephrol Dial Transplant*. 2016;31(5):798-806. doi:10.1093/ndt/gfv454

Reference 2: Van Biesen W, Nagler EV. A Swiss army knife for estimating kidney function: why new equations will not solve the real problem. *Nephrol Dial Transplant*. 2016;31(5):685-687. doi:10.1093/ndt/gfw01

2. *Supplementary Table 1: Genotype of excluded probands harbouring variants in genes associated with other disorders*

Sr. No.	*Gene*	Mutation	Zygosity	Population Frequency (GnomAD/ Topmed)	Prediction tools (SIFT/ PolyPhen2/ LRT/ MutationTaster2, SpliceAI)	Protein Change	ACMG Classification	Disease phenotype	Status (Literature/ databases)
1.	*CA2* NM_000067.3	Intron 4 c.445-1G>C	Homozygous	NP/NP	D- SpliceAI	IVS4-1G>C (splice site)	Likely Pathogenic (PVS1, PM2, PP4)	Osteopetrosis type 3 with RTA	Present study
2.	*SLC7A7* NM_003982.4	Exon 2 c.278T>C	Homozygous	NP/NP	D/PrD/D/D	p.Leu93Pro (Missense)	VUS^ (PM2, PP3)	Lysinuric protein intolerance (LPI)	Present study
3.	*UMOD* NM_003361.4	Exon 4 c.306C>G	Heterozygous	NP/NP	NA	p.Pro102= (Silent)	VUS (PM2, BP7)	Autosomal dominant tubulointerstitial kidney disease (ADTKD)	Present study
4.	*CYP21A2* NM_000500.9	Intron 2 c.293-13C>G	Compound Heterozygous	~0.002/ 0.004	D- SpliceAI	IVS2-13C>G (Splice site)	Pathogenic	Congenital adrenal hyperplasia (21	Reported (PMID:3535

							(PS3, PM2, PM3, PP4)	hydroxylase deficiency)	5919,33604243)
		Exon 7 c.923dup		~0.0002	NA	p.Leu308PhefsTer6 (Frameshift)	Pathogenic (PVS1, PM2, PM3, PP4)		Reported (PMID:35094236,35079965)
5.	*MAGED2* NM_177433.3	Exon 3 c.389C>G	Hemizygous	NP/NP	D/B/B/B	p.Ala130Gly (Missense)	VUS (PM2, BP4)	Transient antenatal Bartter's syndrome	Present study
6.	*AIRE* NM_000383.4	Exon 8 c.967_979del	Compound Heterozygous	~0.0005/ 0.0006	NA	p.Leu323SerfsTer51 (Frameshift)	Pathogenic (PVS1, PM2, PP4, PP5)	Autoimmune polyendocrine syndrome type 1	Reported (PMID:36191466,35753512)
		Exon 10 c.1103dup		0.000006/ 0.000003	NA	p.Leu370AlafsTer2 (Frameshift)	Pathogenic (PVS1, PM2, PP4, PP5)		Reported (PMID:33434148,26141571)
7.	*ATP7B* NM_000053.4	Exon 2 c.813C>A	Heterozygous	0.00003/ 0.000003	NA	p.Cys271Ter (Nonsense)	Pathogenic (PVS1, PM2, PM3)	Wilson's disease	Reported (PMID:3534

									2245,32351182)
8.	*SLC12A1* NM_000338.3	Exon 13 c.1627G>A	Homozygous	NP/NP	PrD/D/D/D	p.Glu543Lys (Missense)	VUS (PM2, PP3, PP4)	Bartter's syndrome type 1	Present study
9.	*SLC12A1* NM_000338.3	Exon 6 c.878T>C	Heterozygous	NP	PrD/D/D/B	p.Leu293Pro (Missense)	VUS (PM2, PP3, PP4)	Bartter's syndrome type 1	Present study
		Exon 13 c.1790C>T	Heterozygous	NP	PrD/D/D/B	p.Thr597Ile (Missense)	VUS (PM2, PP3, PP4)		Present study
10.	*KCNJ1* NM_153766.3	Exon 2 c.658C>T	Homozygous	0.0001/ 0.00003	PrD/D/D/D	p.Leu201Phe (Missense)	Likely Pathogenic (PM2, PM5, PP3, PP4, PP5)	Bartter's syndrome type 2	Present study
11.	*DGAT1* NM_012079.6	Intron 9 c.856-1dup	Homozygous	NP/NP	D- SpliceAI	p.Leu286AlafsTer66 (Splice variant)	Likely Pathogenic (PVS1, PM2, PP4)	Diarrhoea-7, Protein-losing enteropathy type	Present study

D- Damaging, NA- Not applicable, NP- Not present, PrD- Probably Damaging, VUS- Variant of unknown significance

^Alternative variant chr2:240869350 G>T (Gly116Trp) is classified Pathogenic [ClinVar:VCV002681163.1].)

- Parental segregation of variants was observed in proband 4. Not checked for other probands.
- References 39-50 in this paper refer to other similar cases where overlap with dRTA was encountered (Discussion; paragraph 11).

3. Supplementary Table 2: Differences between genotype-positive vs genotype-negative primary dRTA

	Genotype-positive primary dRTA (n=37)	Genotype-negative primary dRTA (n=10)	p value
Baseline characteristics			
Age at symptom-onset (months): Median (IQR)	12 (4-36)	96 (9-312)	0.072
Age at diagnosis (months): Median (IQR)	54 (24-84)	126 (24-348)	0.180
Delay in diagnosis (months): Median (IQR)	27 (3-48)	13.5 (9-36)	0.279
FTT (%)	94.6%	30%	0.000
Polyuria (%)	83.8%	20%	0.000
HKP as presentation (%)	13.5%	50%	0.024
Rickets (%)	91.9%	30%	0.000
Height SDS: Mean (SD)	-4.4 (2.1) (n=25)[#]	-2.2 (5.4) (n=7)[#]	0.318
Serum bicarbonate (meq/L): Mead (SD)	11.5 (3.9) (n=36)[#]	17.7 (3.4)	0.000
Urine pH: Mean (SD)	7.2 (0.7) (n=33)[#]	6.7 (0.6) (n=8)[#]	0.063
eGFR (ml/min/1.73m^2): Mean (SD)	65.7 (23.2) (n=27)[#]	67.2 (30.6) (n=9)[#]	0.875
Hypokalaemia (%)	32/36 (86.5%)	7/9 (77.8%)	0.583
UCCR >0.2 mg/mg (%)	18/26 (69.2%)	100%	0.550

Hypophosphatemia (%)	19/31 (61.3%)	5/9 (55.6%)	1.000
MNC/NL present at diagnosis (%)	23/34 (67.7%)	5/7 (71.4%)	1.000
Follow-up characteristics			
Final Height SDS: Mean (SD)	-3.4 (1.7)	-2.2 (3.7)	0.134
Persistent bony deformities (%)	46%	10%	0.065
First episode of HKP occurring on follow-up (%)	(35.1%)	0	0.043
Recurrent HKP (%)	32.4%	40%	0.716
Bicarbonate supplementation (meq/kg/d): Mean (SD)	3.3 (3.6)	1.4 (1.2) (n=9)#	0.132
Potassium supplementation (meq/kg/d): Mean (SD)	2.5 (3)	1.02 (0.7) (n=7)#	0.218
eGFR at last visit (ml/min/1.73m^2): Mean (SD)	92.2 (25.4)	77.7 (28.4)	0.124
eGFR <90 ml/min/1.73m^2 (%)	54.1%	70%	0.481
eGFR <60 ml/min/1.73m^2 (%)	8.1%	30%	0.101
Proteinuria (%)	24.3%	20%	1.000
MNC (%)	83.8%	20%	0.000
NL (%)	32.4%	60%	0.150

eGFR- estimated glomerular filtration rate, FTT- failure to thrive, HKP- hypokalaemic paralysis, IQR- interquartile range, MNC- medullary nephrocalcinosis, NL- nephrolithiasis, SD- standard deviation, SDS- standard deviation score, UCCR- urinary calcium creatinineratio

#- Number of patients in whom values of the given parameter were available

4. Supplementary Table 3: Phenotypic differences between biallelic forms of primary dRTA (SLC4A1, ATP6V1B1/ATP6V0A4, and WDR72)

	**A. Biallelic *SLC4A1* (n=22)**	**B. *ATP6V1B1/ ATP6V0A4* (n=11)**	**C. *WDR72* (n=9)**	**A vs B**	**A vs C**	**B vs C**
Baseline characteristics						
Age at symptom-onset (months): Median (IQR)	12 (6-24)	3 (2.5-4)	60 (36-72)	0.001	0.001	0.000
Age at diagnosis (months): Median (IQR)	48 (24-84)	6 (3-21)	84 (48-168)	0.001	0.037	0.000
Delay in diagnosis (months): Median (IQR)	28.5 (15-48)	1 (0-10)	27 (12-72)	0.004	0.983	0.067
FTT (%)	21 (95.5%)	10 (90.9%)	6 (66.7%)	1.000	0.063	0.285
Polyuria (%)	18 (81.8%)	8 (72.7%)	7 (77.8%)	0.661	1.000	1.000
HKP as presentation (%)	1 (4.6%)	2(18.2%)	3 (33.3%)	0.252	0.063	0.617
Rickets (%)	18 (81.8%)	8 (72.7%)	7 (77.8%)	0.661	1.000	1.000
Height SDS: Mean (SD)	-4.4 (2.1) (n=14)#	-5.6 (3.3) (n=4)#	-3.6 (1.8) (n=7)#	0.394	0.401	0.223
Serum bicarbonate (meq/L): Mean (SD)	10.6 (4.1) (n=21)#	13.1 (4.2)	13.2 (2.2)	0.121	0.086	0.929

Urine pH: Mean (SD)	7.2 (0.6) (n=20)#	6.6 (0.6) (n=6)#	7.7 (0.5) (n=8)#	0.062	0.027	0.005
eGFR (ml/min/1.73m^2): Mean (SD)	67.3 (28.2) (n=14)#	49.4 (15) (n=4)#	73.1 (9.5) (n=9)#	0.248	0.556	0.005
Hypokalaemia (%)	18/20 (90%)	9 (81.8%)	9(100%)	0.601	1.000	0.479
UCCR >0.2 mg/mg (%)	6/9 (66.7%)	7/9 (77.8%)	5/9 (55.6%)	1.000	1.000	0.620
Hypophosphatemia (%)	10/17 (58.8%)	5/8 (62.5%)	7/9 (77.8%)	1.000	0.418	0.620
Low urinary citrate (%)	13/13 (100%)	7/7 (100%)	4/6 (33.3%)	None	0.088	0.192
MNC detected at first diagnosis (%)	14/19 (73.7%)	7/9 (77.8%)	5/9 (55.6%)	1.000	0.407	0.620
Follow-up characteristics						
Final Height SDS	-3.4 (1.8)	-2.6 (1.3)	-3.4 (2.4)	0.243	0.985	0.390
Persistent bony deformities (%)	9 (40.9%)	3 (27.3%)	4 (44.4%)	0.425	1.000	0.642
First episode of HKP occurring at follow-up (%)	9 (40.9%)	4 (36.4%)	3 (33.3%)	1.000	0.703	1.000
Recurrent HKP (%)	4 (18.2%)	5 (45.5%)	6 (66.7%)	0.555	0.077	1.000
Dose of bicarbonate (meq/kg/d): Mean (SD)	4.4 (5.2)	3.6 (2.7)	1.8 (1.4)	0.640	0.150	0.084

Dose of potassium (meq/kg/d): Mean (SD)	2.2 (1.8) (n=19)#	3.4 (4.8)	1.5 (1) (n=8)#	0.354	0.269	0.281
eGFR (ml/min/1.73m^2): Mean (SD)	91.7 (23.9)	84.6 (23)	103 (33.5)	0.425	0.295	0.163
eGFR <90 ml/min/1.73m^2 (%)	11 (50%)	8 (72.7%)	3 (33.3%)	0.704	0.456	0.175
eGFR <60 ml/min/1.73m^2 (%)	2 (9.1%)	1 (9.1%)	1 (11.1%)	1.000	1.000	1.000
Proteinuria (%)	4 (18.2%)	3 (27.3%)	2 (22.2%)	1.000	1.000	1.000
MNC (%)	16 (72.7%)	10 (90.9%)	8 (88.9%)	0.142	0.639	1.000
NL (%)	7 (31.8%)	0	4 (44.4%)	0.069	0.683	0.026
MNC/NL (%)	20 (90.9%)	10 (90.9%)	9 (100%)	1.000	1.000	1.000
Cysts (%)	4 (18.2%)	1 (9.1%)	4 (44.4%)	1.000	0.185	0.127

eGFR- estimated glomerular filtration rate, FTT- failure to thrive, HKP- hypokalaemic paralysis, IQR- interquartile range, MNC- medullary nephrocalcinosis, NL- nephrolithiasis, SD- standard deviation, SDS- standard deviation score, UCCR- urinary calcium creatinine ratio#- Number of patients in whom values of the given parameter were available

5. Supplementary figure 1: Partial amelogenesis imperfecta (AI) observed in the cohort

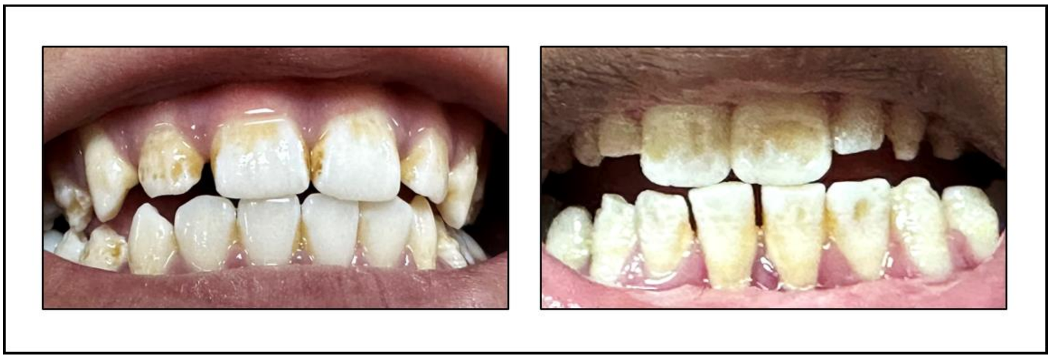

Yellowish discolouration and localised enamel defects noted in two patients in two probands with *SLC4A1*:p.Ala858Asp variant, contrasting with generalised involvement of teeth in patients with amelogenesis imperfecta (AI) type IIA3 observed in association with *WDR72* variant

6. Supplementary table 4: Age at onset of sensorineural hearing deficit (SNHD) in the study cohort (probands and family members)

	ATP6V1B1	**Heterozygous *ATP6V1B1* (VUS)**	***ATP6V0A4***	***FOXI1***	***WDR72***
SNHD (%)	4/7 (57.1%)	1/2 (50%)	3/4 (60%)	1/1 (100%)	1/10 (10%)
Age at onset of SNHD (months)	9, 12, 24, 36	588<br>(mild SNHD only for higher frequencies)	36, 120<br>(Age at onset not available for one proband)	96	84<br>(Incidentally detected in one. Others not evaluated formally as no clinical SHND)

SNHD: Sensorineural hearing deficit

7. Supplementary Table 5: Results of Eosin 5' Maleimide (EMA) dye-binding test for detecting red blood cell osmotic fragility

**Biallelic *SLC4A1***	**Number of patients tested**	**Red cell membrane defect detected**
p.Ala858Asp	13	13
Affected family members of p.Ala858Asp	2	2
Non-affected family members of p.Ala858Asp	3	None
**Monoallelic *SLC4A1***		
p.Arg589Cys	2	None
p.Arg589His	1	None
p.Glu906Gln	1	None
**Others**		
ATP6V1B1	1	None
Genotype-negative	2	None
